# A Small Molecule Inhibitor of PDK1/PLCγ1 Interaction Blocks Breast and Melanoma Cancer Cell Invasion

**DOI:** 10.1038/srep26142

**Published:** 2016-05-20

**Authors:** Claudio Raimondi, Veronique Calleja, Riccardo Ferro, Alessandro Fantin, Andrew M. Riley, Barry V. L. Potter, Caroline H. Brennan, Tania Maffucci, Banafshé Larijani, Marco Falasca

**Affiliations:** 1 Queen Mary University of London, Barts and The London School of Medicine and Dentistry, Blizard Institute, Inositide Signalling Group, London E1 2AT, UK; 2 Cell Biophysics Laboratory, Cancer Research UK, Lincoln’s Inn Fields Laboratories, London Research Institute, London WC2A 3LY, UK; 3 UCL Institute of Ophthalmology, University College London, London EC1V 9EL, UK; 4 Wolfson Laboratory of Medicinal Chemistry, Department of Pharmacy and Pharmacology, University of Bath, Bath BA2 7AY, UK; 5 Department of Pharmacology, University of Oxford, Mansfield Road, Oxford OX1 3QT; 6 Queen Mary University of London, School of Biological and Chemical Sciences, London E1 4NS, UK; 7 Metabolic Signalling Group, School of Biomedical Sciences, Curtin Health Innovation Research Institute, Curtin University, Perth, Western Australia 6102, Australia

## Abstract

Strong evidence suggests that phospholipase Cγ1 (PLCγ1) is a suitable target to counteract tumourigenesis and metastasis dissemination. We recently identified a novel signalling pathway required for PLCγ1 activation which involves formation of a protein complex with 3-phosphoinositide-dependent protein kinase 1 (PDK1). In an effort to define novel strategies to inhibit PLCγ1-dependent signals we tested here whether a newly identified and highly specific PDK1 inhibitor, 2-*O*-benzyl-*myo*-inositol 1,3,4,5,6-pentakisphosphate (2-*O*-Bn-InsP_5_), could affect PDK1/PLCγ1 interaction and impair PLCγ1-dependent cellular functions in cancer cells. Here, we demonstrate that 2-*O*-Bn-InsP_5_ interacts specifically with the pleckstrin homology domain of PDK1 and impairs formation of a PDK1/PLCγ1 complex. 2-*O*-Bn-InsP_5_ is able to inhibit the epidermal growth factor-induced PLCγ1 phosphorylation and activity, ultimately resulting in impaired cancer cell migration and invasion. Importantly, we report that 2-*O*-Bn-InsP_5_ inhibits cancer cell dissemination in zebrafish xenotransplants. This work demonstrates that the PDK1/PLCγ1 complex is a potential therapeutic target to prevent metastasis and it identifies 2-*O*-Bn-InsP_5_ as a leading compound for development of anti-metastatic drugs.

Metastasis, the ability of cancer cells to spread from a primary site and form tumours at distant sites, is the main cause of death in cancer patients^1^. Cancer cells with high metastatic potential are characterised by high proliferation and migration^2^. During metastasis, malignant cells are able to “escape” from the primary tumour through the circulatory or lymphatic system, invading distant healthy tissues to initiate a new malignant lesion. Cell motility is critical for this process to occur and the mechanisms involved in regulation of cell motility are the results of an intricate network of signals activated by different growth factors, chemo-attractant and extracellular matrix (ECM) components *in vivo*^3^.

The endogenous signalling pathways associated with high metastatic potential remain unclear. Strong evidence has demonstrated that activation of the enzyme phospholipase Cγ1 (PLCγ1) is a key and crucial step in metastasis development and progression^3^,^4^,^5^,^6^. PLCγ1 is activated by integrin engagement to the ECM and it also plays an important role in growth factor-induced cell motility, being activated by tyrosine kinase receptors such as epidermal growth factor (EGF) receptor^3^,^5^,^6^. Recent evidence produced by our group confirmed the pivotal role of PLCγ1 in malignant cell invasion and its role in metastasis development and progression^4^. PLCγ1 possesses a pleckstrin homology (PH) domain that can bind to the phosphoinositide 3-kinase (PI3K) lipid product phosphatidylinositol 3,4,5-trisphosphate [PtdIns(3,4,5)*P*_*3*_] and contribute to translocation of the enzyme to the plasma membrane^7^. Our previous work demonstrated that this binding is essential for PLCγ1 activation^7^ and it is required for EGF-induced migration of breast cancer cells^8^ and basic fibroblast growth factor-mediated migration and remodelling of human umbilical vein endothelial cells^9^.

Deregulated PI3K signalling is very common in cancer and it leads to dysregulation of several intracellular processes, including cell survival, growth, proliferation and migration^10^,^11^. Among the downstream effectors of PI3Ks, 3-phosphoinositide-dependent protein kinase 1 (PDK1) and AKT have key roles in several cancer types^12^. PDK1 in particular is highly expressed in many human breast cancer cell lines and both PDK1 protein and mRNA are overexpressed in a majority of human breast cancers^13^,^14^. Overexpression of PDK1 is sufficient to transform mammary epithelial cells and, furthermore, PDK1 has an essential role in regulating cell migration^15^,^16^. Moreover, we have recently demonstrated that PDK1 can regulate PLCγ1 activation in a mechanism involving assembly of a complex between the two enzymes upon growth factor stimulation^17^.

Similar to PLCγ1, PDK1 possesses a PH domain and, in addition to the phosphoinositide PtdIns(3,4,5)*P*_3_, the PH domain of PDK1 has been shown to bind inositol phosphates such as inositol 1,3,4,5-tetrakisphosphate [Ins(1,3,4,5)*P*_4_] and inositol 1,3,4,5,6-pentakisphosphate (InsP_5_)^18^. Interestingly, InsP_5_ exhibits anti-angiogenic properties and it induces apoptosis in ovarian, lung and some breast cancer cell lines^19^,^20^ by preventing AKT phosphorylation. We have recently identified an InsP_5_ derivative, 2-*O*-benzyl-*myo*-inositol 1,3,4,5,6-pentakisphosphate (2-*O*-Bn-InsP_5_), that possesses higher pro-apoptotic activity than InsP_5_^21^. Kinase profiling assay revealed that 2-*O*-Bn-InsP_5_ specifically inhibits PDK1 *in vitro* in the low nanomolar range (IC_50_ = 26 nM). Importantly, we demonstrated that this compound was able to inhibit PDK1-dependent phosphorylation of AKT in cell lines and excised tumours^21^.

Here we investigated the effect of 2-*O*-Bn-InsP_5_ on the novel identified PDK1/PLCγ1 signalling pathway and its mechanism of action. We show that 2-*O*-Bn-InsP_5_ binds to the PH domain of PDK1 with high affinity, as assessed by isothermal titration calorimetry (ITC). By binding PDK1 PH domain, 2-*O*-Bn-InsP_5_ prevents the formation of the PDK1/PLCγ1 complex. This in turn results in inhibition of cell migration, 3D Matrigel invasion of breast and melanoma cell lines *in vitro*, and ultimately tumour cell dissemination in zebrafish xenotransplants.

Taken together these data demonstrate that targeting the PDK1/PLCγ1 is a promising strategy to prevent metastatic dissemination. Furthermore, these data identify 2-*O*-Bn-InsP_5_ as a leading compound to develop anti-metastatic drugs.

## Results

### 2-*O*-Bn-InsP_5_ is a specific PDK1 PH domain inhibitor

**Figure 1:**
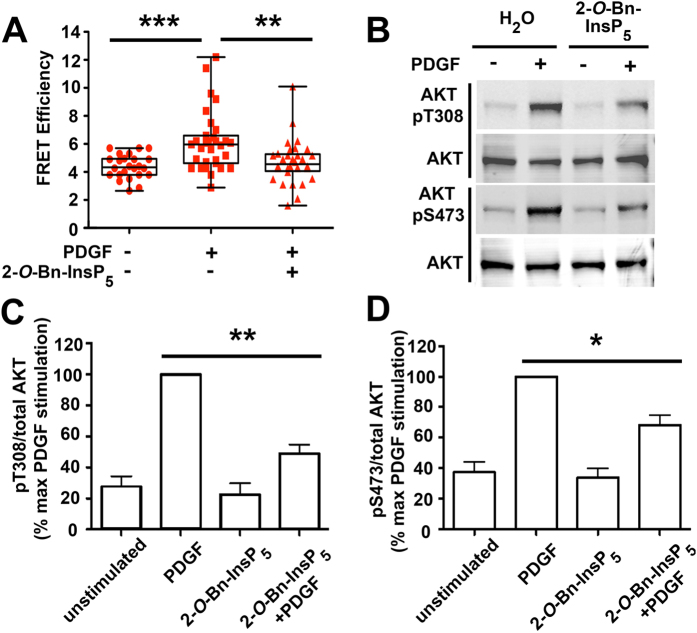
PDGF-induced change in conformation and phosphorylation of AKT are inhibited upon treatment with 2-*O*-Bn-InsP_5_. (**A**) The increase in FRET efficiency upon PDGF treatment indicates a change in conformation of AKT that is prevented upon pre-treatment with the inhibitor. Box and whiskers plots of FRET efficiencies are displayed for the indicated conditions. Each cell is represented by a red symbol. A Mann-Whitney test was used to calculate the *P* values shown on the graph (n = 3 experiments; **p = 0.0016, ***p = 0.0001). (**B**) Cells were left untreated or treated with 2-*O*-Bn-InsP_5_ prior to PDGF stimulation and phosphorylation of AKT at residues Threonine 308 (pT308) and Serine 473 (pS473) was assessed by Western blotting. (**C**,**D**) Results from densitometry analysis of Western blotting showing significant decrease in the normalized phosphorylation of AKT at T308 and S473 from 4 independent experiments (**p = 0.002, *p = 0.007 respectively).

We have recently identified a synthetic derivative of the natural compound InsP_5_, named 2-*O*-Bn-InsP_5_, which potently and specifically inhibits PDK1 *in vitro* as well as the PDK1-dependent phosphorylation of AKT Thr308 in cancer cell lines and *in vivo*^21^. To further characterise the anti-PDK1 activity of this compound we first determined whether 2-*O*-Bn-InsP_5_ was able to interfere with the platelet-derived growth factor (PDGF)-induced, PDK1-dependent AKT activation. To this end, NIH 3T3 cells were pre-treated for 10 min with 2-*O*-Bn-InsP_5_ followed by 5 minutes stimulation with 30 ng/ml of PDGF (Fig. 1A) and changes in AKT conformation were monitored by a time-resolved FRET approach using an AKT activation sensor (eGFP-AKT-mRFP). Data revealed that PDGF treatment induced a conformational change in eGFP-AKT-mRFP and this was inhibited by pre-treating the cells with 2-*O*-Bn-InsP_5_ (Fig. 1A). As a result of this impaired conformational change, 2-*O*-Bn-InsP_5_ strongly inhibited the PDGF-induced phosphorylation of AKT at its residues Thr308 and Ser473 in NIH 3T3 transfected with eGFP-PDK1 and eGFP-AKT (Fig. 1B–D). Taken together, these data demonstrate that 2-*O*-Bn-InsP_5_ inhibits PDK1-dependent AKT phosphorylation and are consistent with our previous data *in vitro* and *in vivo*^21^.

**Figure 2:**
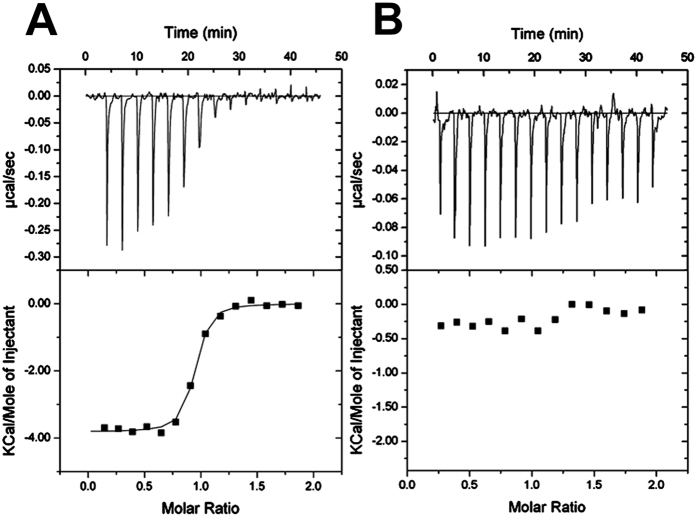
2-*O*-Bn-InsP_5_ binds to the PH domain of PDK1 but not that of AKT2. (**A**) Titration of 2-*O*-Bn-InsP_5_ into the PH domain of PDK1 results in a decrease in binding heats as the titration proceeds (top panel) giving a distinct binding isotherm (bottom panel). (**B**) Titration of 2-*O*-Bn-InsP_5_ into the PH domain of AKT2 did not show decrease in binding during titration analysis. Heats corresponding to each injection are small and remain constant and are equivalent to the heats of dilution indicating that no binding occurs.

Since it has been reported that InsP_5_ can bind to PDK1 PH domain we hypothesised that 2-*O*-Bn-InsP_5_ could inhibit PDK1 by binding to this domain and therefore interfere with its interaction with PtdIns(3,4,5)*P*_*3*_. To test this hypothesis, we determined the thermodynamic parameters of 2-*O*-Bn-InsP_5_ binding to PDK1 PH domain by ITC (Table 1). The closely related PH domain of AKT2 was used as a control to monitor the specificity of binding of the inhibitor (Supplementary Figure 1A). From four independent experiments, performed at 25 °C, the binding affinity of 2-*O*-Bn-InsP_5_ for the PH domain of PDK1 (*K*_d_) was calculated to be 109 ± 44 nM with a 1:1 stoichiometry (n = 0.90 ± 0.04) (Table 1 and Fig. 2A). Binding was driven by a favourable enthalpic contribution ΔH_obs_ = −4.1 ± 0.5 kcal/mol and an entropic (−TΔS) contribution of −0.291 ± 0.002 kcal/mol. However, no significant heat change was detected by titrating the inhibitor into the AKT2 PH domain performed at 25 °C (Fig. 2B) and at 15 °C indicating that the inhibitor is specific for the PDK1 PH domain. The specificity of 2-*O*-Bn-InsP_5_ for PDK1 is consistent with our previous results from a kinase profiling assay that tested ~50 distinct kinases *in vitro* and that revealed a selective inhibition of PDK1 activity^21^. Importantly no direct inhibition of AKT activity *in vitro* was detected in this assay^21^, consistent with ITC results.

To further assess the specificity of 2-*O*-Bn-InsP_5_ in a cellular context, MDA-MB-231 cells were treated with 2-*O*-Bn-InsP_5_ 50 µM or vehicle alone before stimulation with EGF and activation of several enzymes was assessed using a commercially available Proteome Profiler Human Phospho-Kinase Array Kit. Out of 45 proteins tested, 2-*O*-Bn-InsP_5_ treatment specifically affected only the EGF-induced phosphorylation of AKT, ERK1/2 and PLCγ1 (Supplementary Figure 1B), confirming the high specificity of this compound. Specifically, 2-*O*-Bn-InsP_5_ reduced the PDK1-dependent phosphorylation of AKT at its residues Thr308 and Ser473 (Supplementary Figure 1B), consistent with our previous data (Fig. 1B–D) and our previous report^21^. 2-*O*-Bn-InsP_5_ reduced phosphorylation of PLCγ1 at its residue Tyr783 (Supplementary Figure 1B), in agreement with our previous study demonstrating that chemical inhibition of PDK1 was able to affect the EGF-induced Tyr783 PLCγ1 phosphorylation^17^. Validation of the Proteome Profiler Human Phospho-Kinase Array data by immunoblotting analysis confirmed reduced EGF-induced AKT phosphorylation but similar ERK1/2 phosphorylation in MDA-MB-231 treated with 2-*O*-Bn-InsP_5_ compared to cells treated with vehicle only, suggesting that 2-*O*-Bn-InsP_5_ does not affect ERK1/2 phosphorylation (Supplementary Figure 1C). Taken together these data strongly suggest that 2-*O*-Bn-InsP_5_ specifically inhibits PDK1 activation in a mechanism involving its binding to PDK1 PH domain.

### 2-*O*-Bn-InsP_5_ inhibits PLCγ1-PDK1 complex formation in MDA-MB-231 breast cancer cells

**Figure 3:**
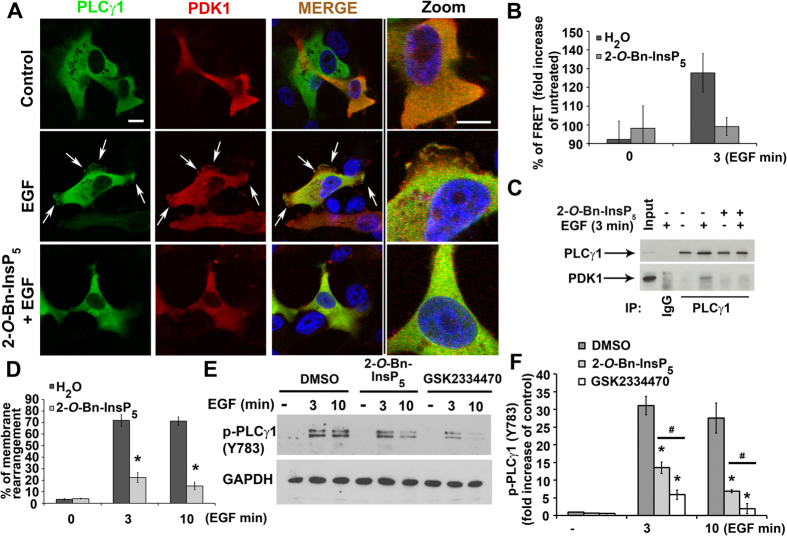
2-*O*-Bn-InsP_5_ inhibits PLCγ1-PDK1 complex formation and PLCγ1 tyrosine phosphorylation in MDA-MB-231 cells. (**A**) Localisation of PLCγ1 and PDK1 visualised by high definition confocal scan in MDA-MB-231 overexpressing PLCγ1 and PDK1. Cells were left untreated or treated with 2-*O*-Bn-InsP_5_ 50 μM and then stimulated with EGF for 3 min. Arrows indicate plasma membrane localisation. Bar: 20 μm. (**B**) Percentage of FRET in MDA-MB-231 cells co-expressing PLCγ1 and PDK1 at the indicated time of EGF stimulation. Columns are the means ± SEM of percentage of FRET measured at protrusion level in proximity of the plasma membrane. (**C**) Endogenous PLCγ1 and PDK1 were co-immunoprecipitated from MDA-MB-231 untreated or treated with 2-*O*-Bn-InsP_5_ 50 μM and stimulated with EGF 50 ng/ml for the indicated time points. PLCγ1 was immunoprecipitated using a specific anti-PLCγ1 antibody and association of the two enzymes was assessed by immunoblotting using anti-PLCγ1 and anti-PDK1 antibodies. Parallel co-immunoprecipitation using mouse IgG was performed as control. (**D**) Percentage of MDA-MB-231 cells displaying plasma membrane rearrangement presenting both PLCγ1 and PDK1 staining at the plasma membrane. Cells expressing exogenous PLCγ1 and PDK1 were stimulated with EGF for the indicated times in the presence or absence of 2-*O*-Bn-InsP_5_ 50 μM. Graph shows the means ± SEM from n = 3 independent experiments. (**E**,**F**) Representative Western blot (**E**) and corresponding densitometry analysis (**F**) of EGF-induced PLCγ1 tyrosine phosphorylation in MDA-MB-231 untreated or treated with 2-*O*-Bn-InsP_5_ 50 μM or GSK2334470 1 μM. In E, GAPDH was used as loading control. Data in F are means ± SEM from n = 4 independent experiments. *P* value: *<0.05; ^#^≤0.01.

It is well established that PDK1 is required for activation of several AGC kinases including AKT. More recently we demonstrated that PDK1 can also regulate activation of PLCγ1 in a mechanism involving formation of a specific complex between the two enzymes^17^. We therefore decided to investigate whether inhibition of PDK1 by 2-*O*-Bn-InsP_5_ might affect the formation of PLCγ1/PDK1 protein complex. Stimulation of MDA-MB-231 overexpressing PLCγ1 and PDK1 with EGF for 3 minutes induced major reorganisation of the plasma membrane (Fig. 3A white arrows) and formation of a PDK1/PLCγ1 complex, as demonstrated by increased steady-state FRET signal (Fig. 3B), as previously reported^17^. Treatment of MDA-MB-231 with 2-*O*-Bn-InsP_5_ inhibited reorganisation of the plasma membrane (Fig. 3A) and completely blocked the EGF-induced FRET signal (Fig. 3B) indicating that 2-*O*-Bn-InsP_5_ prevents the formation of the PLCγ1/PDK1 complex (Fig. 3B). In agreement with results from FRET analysis, treatment with 2-*O*-Bn-InsP_5_ also reduced the EGF-induced endogenous association between PLCγ1 and PDK1 detected by immunoprecipitation analysis in MDA-MB-231 (Fig. 3C). Quantification of the number of cells showing major membrane rearrangements and localisation of both PLCγ1 and PDK1 at the plasma membrane confirmed that treatment with 2-*O*-Bn-InsP_5_ strongly inhibited the EGF-induced translocation of both enzymes (Fig. 3D).

Taken together these data demonstrate for the first time that 2-*O*-Bn-InsP_5_ inhibits association of PLCγ1 and PDK1 at the plasma membrane.

### 2-*O*-Bn-InsP_5_ inhibits the EGF-induced PLCγ1 tyrosine phosphorylation

We previously reported that the formation of a PDK1/PLCγ1 complex is involved in regulation of PLCγ1 activation^17^. Importantly, data from the phosphokinase array indicated that treatment with 2-*O*-Bn-InsP_5_ reduced the EGF-induced phosphorylation of PLCγ1 at its residue Tyr783 (Supplementary Figure 1B), which is required for the enzyme activation. To better investigate whether the effect of 2-*O*-Bn-InsP_5_ on PDK1/PLCγ1 complex formation results in inhibition of PLCγ1 activity, MDA-MB-231 cells were left untreated or treated with 2-*O*-Bn-InsP_5_ before stimulation with EGF (Fig. 3E,F). The PDK1 inhibitor GSK2334470 was used in parallel in these experiments. Consistent with our previous results^17^, treatment with GSK2334470 strongly reduced the EGF-induced PLCγ1 phosphorylation (Fig. 3E,F). Importantly, treatment with 2-*O*-Bn-InsP_5_ also significantly inhibited PLCγ1 phosphorylation upon EGF stimulation (Fig. 3E,F, Supplementary Figure 1C). These data indicate for the first time that blockade of the PDK1/PLCγ1 complex formation by 2-*O*-Bn-InsP_5_ is able to inhibit Tyr783 PLCγ1 phosphorylation.

### 2-*O*-Bn-InsP_5_ inhibits the EGF-induced calcium release in MDA-MB-231 and MDA-MB-435 breast cancer cells

**Figure 4:**
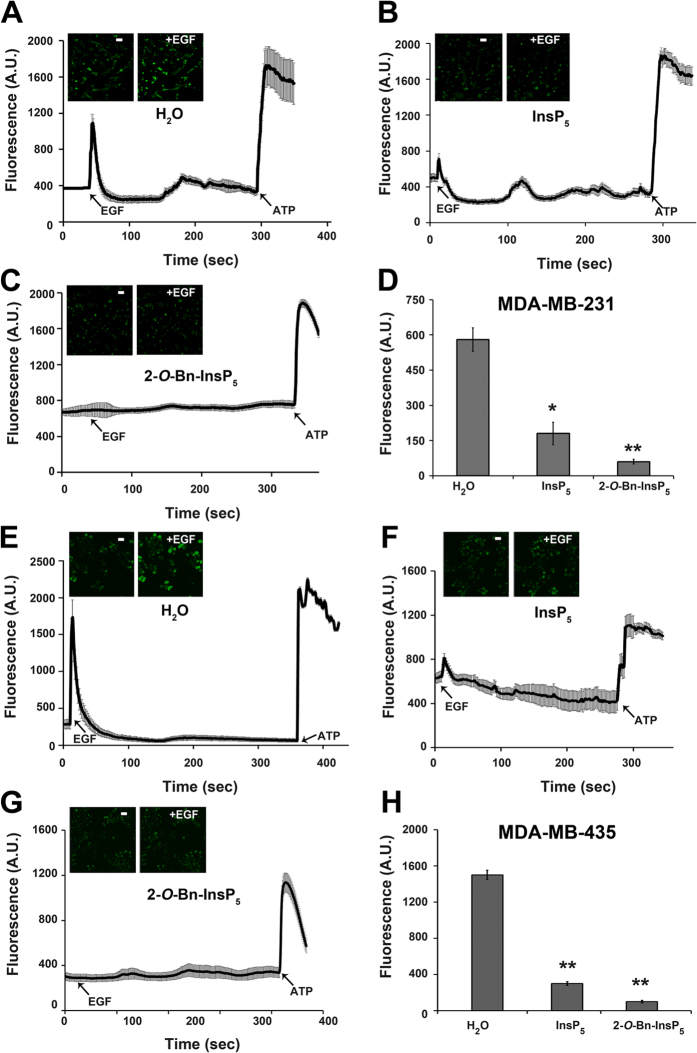
2-*O*-Bn-InsP_5_ inhibits EGF-induced calcium release in MDA-MB-231 and MDA-MB-435 cells. Calcium release assay performed in MDA-MB-231 (**A**–**D**) and MDA-MB-435 (**E**–**H**) treated with 2-*O*-Bn-InsP_5_ and InsP_5_ (50 μM). Arrows indicate the time point(s) of EGF and ATP stimulation. The chart is the mean ± SEM of 4 independent experiments; asterisks indicate *P* value; *<0.05; **≤0.01.

To further investigate the effect of 2-*O*-Bn-InsP_5_ on PLCγ1 signalling we next determined the effect of this compound on EGF-induced calcium release, a process dependent on PLCγ1 activation. The parental molecule InsP_5_ which has a higher IC_50_ for PDK1 (613 nM) compared to 2-*O*-Bn-InsP_5_ (26.5 nM)^21^, was used in parallel in these experiments. Consistent with our previous results^17^, stimulation of MDA-MB-231 and MDA-MB-435 cells with EGF induced a clear PLCγ1-dependent increase of intracellular calcium levels (Fig. 4A,E). Importantly, treatment with InsP_5_ significantly reduced the EGF-induced calcium release in MDA-MB-231 (Fig. 4B,D) and in MDA-MB-435 (Fig. 4F,H). Furthermore treatment with 2-*O*-Bn-InsP_5_ completely suppressed the EGF-induced intracellular calcium increase in MDA-MB-231 (Fig. 4C,D) and MDA-MB-435 (Fig. 4G,H).

These data demonstrate for the first time that 2-*O*-Bn-InsP_5_ inhibits the EGF-induced PLCγ1 activation and confirmed the higher inhibitory activity of 2-*O*-Bn-InsP_5_ compared to the parental molecule (Fig. 4).

### 2-*O*-Bn-InsP_5_ inhibits fibronectin-induced cell migration in MDA-MB-231

**Figure 5:**
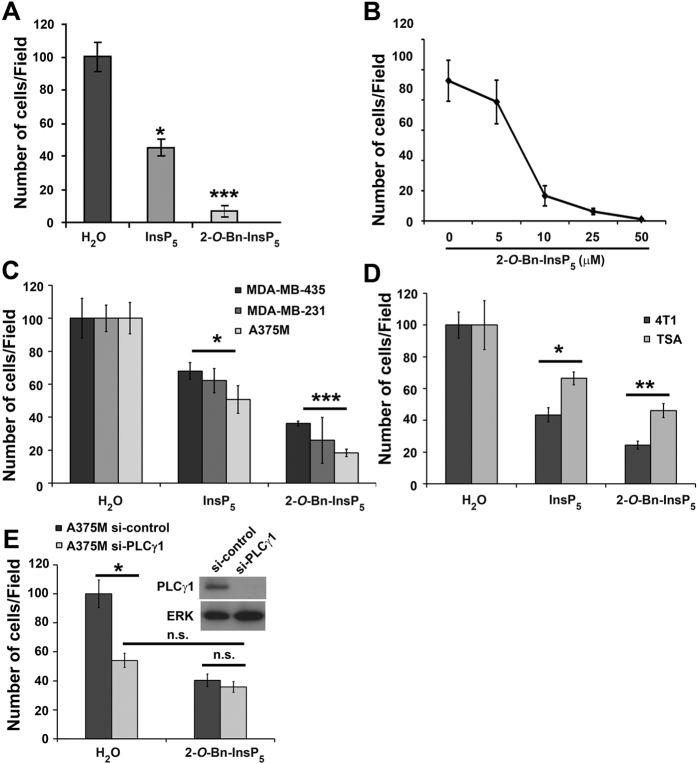
2-*O*-Bn-InsP_5_ inhibits cell migration in different cell lines. (**A**) Results from fibronectin-induced migration of MDA-MB-231 cells assessed by Transwell assays in the presence of 2-*O*-Bn-InsP_5_ or InsP_5_ (50 μM). Control cells were treated with vehicle alone. (**B**) MDA-MB-231 cells were pre-treated with the indicated concentrations of 2-*O*-Bn-InsP_5_. Control cells were pre-treated with H_2_O as control. Fibronectin-induced migration of MDA-MB-231 cells was assessed by Transwell assays. (**C,D**) Results from invasion of the indicated cancer cell lines assessed using transwell inserts pre-coated with Matrigel. Cells were treated with 2-*O*-Bn-InsP_5_, InsP_5_ (50 μM) or vehicle control. (**E**) Invasion of A375M transfected with siRNA targeting PLCγ1 (si-PLCγ1) or with a control non-targeting siRNA (si-control) untreated or treated with 2-*O*-Bn-InsP_5_ 50 μM. In all panels data indicate the number of migrated or invaded cells/field and are expressed as percentage of control, vehicle-treated cells. Data are means ± SEM from n = 3 independent experiments. *p≤0.01; **p≤0.003; ***p≤0.001.

Our previous study demonstrated that the PDK1/PLCγ1 complex is required for cancer cell migration and invasion^17^. Therefore, we next investigated whether inhibition of the complex formation and PLCγ1 activation by 2-*O*-Bn-InsP_5_ was able to affect migration of MDA-MB-231 cells. The effect of the parental molecule InsP_5_ was also determined in these experiments. Results from transwell migration assays revealed that 2-*O*-Bn-InsP_5_ completely blocked fibronectin-induced cell migration in MDA-MB-231 (Fig. 5A). Importantly, 2-*O*-Bn-InsP_5_ had a more potent inhibitory effect than InsP_5_ when tested at the same concentration (50 μM) (Fig. 5A). Dose response analysis showed that 2-*O*-Bn-InsP_5_ was able to inhibit cell migration by 80% compared to untreated cells at a concentration as low as 10 μM (Fig. 5B). These data demonstrate that 2-*O*-Bn-InsP_5_ inhibits cancer cell migration.

### 2-*O*-Bn-InsP_5_ inhibits cell invasion on Matrigel in different human and murine breast cancer cell lines

We next investigated the effect of 2-*O*-Bn-InsP_5_ on cancer cell invasion on Matrigel. Invasion assays were performed using MDA-MB-231 human breast cancer cell line, MDA-MB-435 and A375M human melanoma cell lines (Fig. 5C) and murine syngeneic breast cancer cell lines TSA and 4T1 (Fig. 5D). Data showed that 2-*O*-Bn-InsP_5_ strongly inhibits Matrigel invasion in all cell lines tested. In particular, 52.7%, 63.8%, 73.9% and 87.9% inhibition was observed in TSA, MDA-MB-435, MDA-MB-231 and 4T1 cells, respectively. Consistent with data on migration, 2-*O*-Bn-InsP_5_ inhibited cell invasion on Matrigel more potently than InsP_5_ (Fig. 5C,D). To investigate whether 2-*O*-Bn-InsP_5_ inhibits cancer cell Matrigel invasion by specifically inhibiting PDK1/PLCγ1 pathway, we performed invasion assays using A375M cells transfected with anti-PLCγ1 specific siRNA (si-PLCγ1) or a non-targeting siRNA sequence (si-control) and treated with 2-*O*-Bn-InsP_5_ or with vehicle only (Fig. 5E). Downregulation of PLCγ1 reduced A375M invasion on Matrigel compared to control cells (Fig. 5E), consistent with our previous report^17^. Importantly, we observed that while treatment with 2-*O*-Bn-InsP_5_ reduced invasion in cells transfected with control siRNA it did not further reduce invasion in cells transfected with the siRNA targeting PLCγ1 (Fig. 5E), indicating that 2-*O*-Bn-InsP_5_ treatment has no additive inhibitory effect on cell invasion in cells lacking PLCγ1. Taken together these data demonstrate that 2-*O*-Bn-InsP_5_ is able to inhibit invasion of several cancer cell lines more efficiently than the parental molecule InsP_5_.

### 2-*O*-Bn-InsP_5_ does not affect cell proliferation and cell survival in MDA-MB-435, 4T1 and TSA cell lines

We next assessed the effect of 2-*O*-Bn-InsP_5_ on cell proliferation and cell survival. Cell counting assays showed that treatment with either 2-*O*-Bn-InsP_5_ or InsP_5_ for 72 hours did not affect cell proliferation or cell survival of MDA-MB-231, MDA-MB-435 and TSA cell lines (Supplementary Figure 2A–C). Similarly, no change in proliferation was observed in 4T1 cells treated with 2-*O*-Bn-InsP_5_ (Supplementary Figure 2D). Interestingly a decreased number of 4T1 cells was detected upon treatment with 2-*O*-Bn-InsP_5_ in serum free conditions.

### 2-*O*-Bn-InsP_5_ inhibits tumour cell dissemination in zebrafish xenotransplant

**Figure 6:**
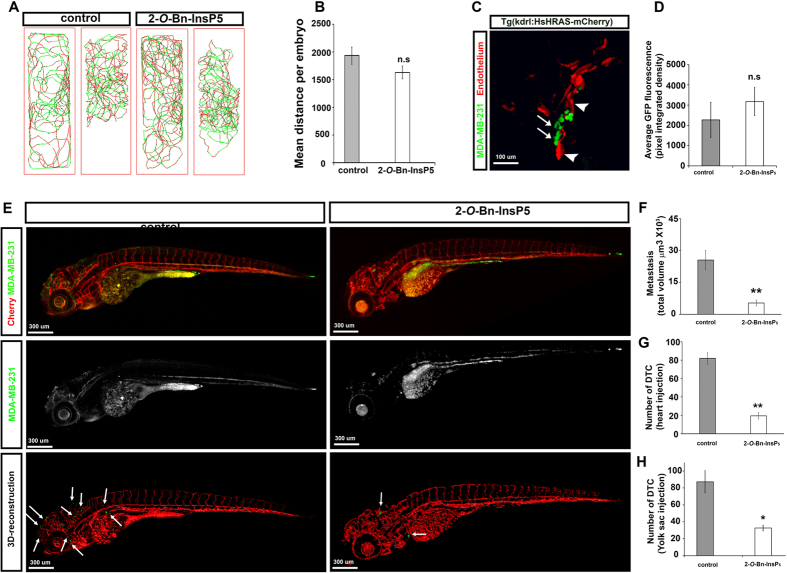
2-*O*-Bn-InsP_5_ inhibits MDA-MB-231 cells dissemination in zebrafish xenotransplant. (**A**) Representative tracks showing the swimming behaviour of 6dpf embryos, untreated or treated with 2-*O*-Bn-InsP_5_ (100 μM) and corresponding quantification (**B**) of the locomotor activity measured. Graph shows the average of the total distance ± SEM covered by each embryos (n = 15 untreated; n = 16 treated). (**C**) Representative image of 48 h post fertilisation *Tg(kdrl:HsHRAS-mCherry)*^*s896*^ zebrafish embryos injected with MDA-MB-231 cells stably expressing GFP. Embryos express Cherry fluorescent protein specifically in endothelial cells. Arrows indicate the injected cancer cells into the cardiac chamber. Arrowheads indicate the heart. (**D**) Zebrafish embryos injected with MDA-MB-231 and treated with or without 2-*O*-Bn-InsP_5_ (100 μM) immunostained using anti-Cherry and anti-GFP antibodies and imaged by high resolution confocal microscopy. GFP-derived fluorescence was quantified in maximal z-stack projections of untreated and treated embryos (untreated n = 7; treated n = 7). (**E**) Image shows maximal z-stack projections and 3D-rendered surface of the vasculature (red) of zebrafish embryos and metastasis (green) in control and treated embryos. Implantation of MDA-MB-231 cells in zebrafish embryos resulted in widespread tumour cell dissemination and metastasis at 5dpf (white arrows). (**F**,**G**) The volume of metastasis and number of disseminated tumour cells (DTC) were evaluated using Imaris. Graphs indicate means ± SEM from n = 6 control and n = 10 treated embryos. Asterisks indicate **P value ≤ 0.01. (**H**) MDA-MB-231 cells stably expressing GFP were injected into the perivitelline cavity of 48 h zebrafish embryos. 2-*O*-Bn-InsP_5_ (100 μM) was added 3 h after injection into the marine salt water. Graph shows the number of DTC counted at 5dpf and indicates means±SEM from n = 10 control and n = 7 treated embryos.

To analyse whether 2-*O*-Bn-InsP_5_ is able to inhibit the dissemination of breast cancer cells *in vivo*, we used a model of xenotransplant in zebrafish embryos. First, we tested 2-*O*-Bn-InsP_5_ for toxic effect on zebrafish. Importantly, no toxic effects were observed when zebrafish embryos were treated with 100 μM 2-*O*-Bn-InsP_5_ as judged by comparing movement (Fig. 6A) and total distance (Fig. 6B) covered by untreated and 2-*O*-Bn-InsP_5_-treated larvae at day 6dpf.

Then, we injected highly metastatic MDA-MB-231 breast cancer cells stably expressing GFP into the heart^22^ of 48h post fertilization *Tg(kdrl:HsHRAS-mCherry)*^*s896*^ zebrafish embryos, which express Cherry fluorescent protein specifically in endothelial cells. To assess the correct injection of tumour cells into the heart and/or cardiac chamber, zebrafish embryos were live-imaged by confocal microscopy (Fig. 6C) immediately after the injection. Embryos displaying a similar number and distribution of injected tumour cells were selected and randomly divided into a group that was left untreated and a group that was treated with 2-*O*-Bn-InsP_5_ 100 μM. Then, after three days, zebrafish embryos were fixed, immunostained using anti-Cherry and anti-GFP antibodies and imaged by high resolution confocal microscopy. Embryos untreated or treated with 2-*O*-Bn-InsP_5_ showed a similar amount of GFP-derived fluorescence (Fig. 6D). Because GFP-derived fluorescence is proportional to the number of GFP-expressing MDA-MB-231 cells injected, this data indicates that untreated and treated embryos had a similar number of surviving cancer cells. Injected embryos untreated or treated with 2-*O*-Bn-InsP_5_ were imaged by high resolution confocal microscopy and Z-stack scans were used to visualise cherry-positive blood vessels and GFP-positive MDA-MB-231 (Fig. 6E top and middle panels). Then, we used Imaris software to generate 3D-rendered projections of the embryos vasculature and to exclude background green fluorescence observed in the yolk sack and gut before quantifying metastases number and volume (Fig. E bottom panels). Implantation of MDA-MB-231 breast cancer cell in zebrafish embryos resulted in widespread tumour cell dissemination and metastasis at 5dpf (Fig. 6E–G). Treatment with 2-*O*-Bn-InsP_5_ significantly reduced the average volume of metastasis compared to untreated embryos (Fig. 6E,F) and the number of disseminated metastasis (Fig. 6E,G). Similar results were obtained when GFP-expressing MDA-MB-231 cells were injected in the perivitelline cavity of 48 h post fertilization embryos (Fig. 6H). These results demonstrate for the first time that 2-*O*-Bn-InsP_5_ prevents MDA-MB-231 cells from disseminating and support the conclusion that inhibition of the novel PDK1/PLCγ1 pathway may represent a novel anti-metastatic strategy.

## Discussion

Several lines of evidence from different groups including our own have indicated that inhibition of PLCγ1 may represent a promising strategy to block metastasis spread. In particular, we previously demonstrated that downregulation of PLCγ1 expression was able to revert metastasis formation in nude mice^4^. These data strongly supported the conclusion that PLCγ1 inhibition has therapeutic potential to counteract metastasis dissemination and growth^4^. Despite this evidence and further data indicating that PLCγ1 has a key role in tumourigenesis^3^,^6^,^12^, the development of selective inhibitors has proven problematic since phospholipases in general are not very good pharmacological targets^6^,^23^. In our quest for inhibitors of PLCγ1 signalling we devised a strategy based on defining the signalling pathway and the interacting partners of PLCγ1 rather than specifically targeting this phospholipase. This led us to the discovery for PDK1 as a novel PLCγ1 interacting protein^17^ involved in the activation of the phospholipase and in the regulation of PLCγ1-dependent cellular functions, including cell invasion. Consistent with a role for PDK1 in tumorigenesis, PDK1 overexpression and increased copy number in breast cancer has been shown to correlate with upstream lesions such as PTEN loss, PIK3CA mutation and EGFR amplification, and resulted in increased tumour growth, cell motility and poor prognosis^13^. Our previous study further suggests a major role for PDK1 in controlling metastasis development and progression by promoting PLCγ1 activation^17^, strengthening the notion that PDK1 is an important potential target to develop novel anti-cancer strategies^12^,^14^. In this scenario the novel PDK1/PLCγ1 interaction might play an important role and could be an important therapeutic target for metastasis.

We anticipated the targeting of PDK1/PLCγ1 interaction at the plasma membrane to be an efficient strategy to counteract PLCγ1 activation. Therefore, we aimed at designing an allosteric inhibitor that would specifically impair PDK1 plasma membrane localisation (rather than targeting its kinase domain) by interfering with its PH domain binding. We predicted that this approach would also lead to the finding of more tumour-specific and less toxic inhibitors. Structural analysis determined that PDK1 PH domain has an unusually spacious ligand binding site compared to other PtdIns(3,4,5)*P_3_*-binding PH domains^18^. In particular, PDK1 PH domain presents additional space around the D2- and D6-hydroxyl groups, which potentially could accommodate further phosphate groups. Thus, in addition to binding the PtdIns(3,4,5)*P_3_* head group, PDK1 PH domain could also bind to the soluble inositols InsP_5_ and InsP_6_. 2-*O*-Bn-InsP_5_ was designed to exploit the peculiar PDK1-PH domain structure in order to reach high specificity. ITC experiments show that 2-*O*-Bn-InsP_5_ binds to PDK1-PH domain but not to AKT2-PH domain suggesting that 2-*O*-Bn-InsP_5_ inhibits specifically PDK1 activation by direct binding to its PH domain (Fig. 2). In agreement, PDK1 is inhibited with high selectivity by 2-*O*-Bn-InsP_5_ in a SelectScreen kinase profiling service^21^.

**Figure 7:**
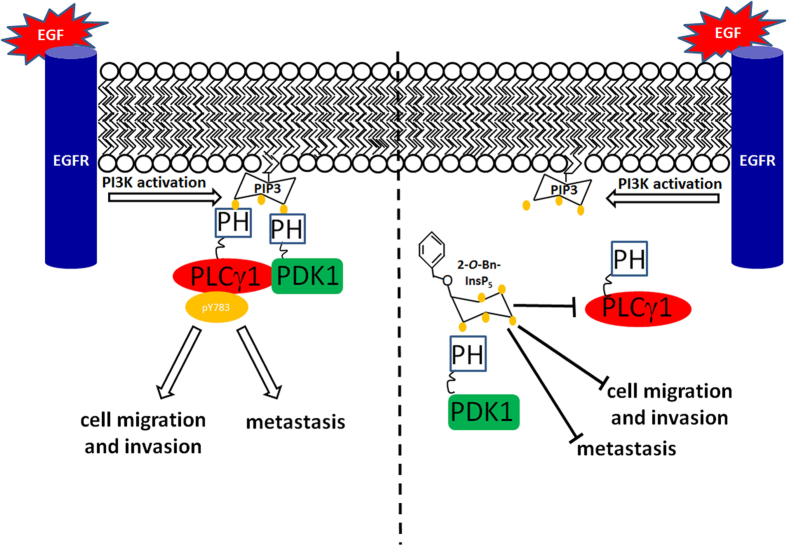
Schematic representation of the potential mechanism by which 2-*O*-Bn-InsP_5_ inhibits the PDK1-dependent PLCγ1 activation. EGF induces phosphorylation of EGF receptor and activation of PI3Ks which catalyse the synthesis of PtdIns(3,4,5)*P*_3_ (PIP3). PIP3 in turn recruits the protein complex PDK1/PLCγ1 to the plasma membrane where PLCγ1 is activated in a mechanism involving phosphorylation of its residue Tyr783. Recruitment of the protein complex and PLCγ1 activation stimulate cell migration, invasion and metastasis dissemination. By competing with PIP_3_ for the binding to PDK1 PH domain, 2-*O*-Bn-InsP_5_ prevents the formation of PDK1/PLCγ1 complex and the recruitment of both proteins to the plasma membrane. Thus 2-*O*-Bn-InsP_5_ inhibits PLCγ1 phosphorylation and activity and reduces tumour cell migration and metastasis dissemination. Yellow dots are phosphate groups bound to the inositol ring.

Here we show for the first time that 2-*O*-Bn-InsP_5_ targets the recently discovered PDK1-dependent PLCγ1 activation^17^ by impairing PDK1 and PLCγ1 plasma membrane localisation and the formation of the PDK1/PLCγ1 complex required for the PDK1-dependent PLCγ1 phosphorylation and activation (Figs 3 and 7). 2-*O*-Bn-InsP_5_ treatment reduced PDK1/PLCγ1 complex formation as shown by FRET and co-immunoprecipitation analyses (Fig. 3). More importantly, we show that the 2-*O*-Bn-InsP_5_-mediated inhibition of PDK1/PLCγ1 complex assembly results in inhibition of cancer cell migration, invasion and *in vivo* dissemination using zebrafish xenotransplants (Fig. 6). Together these results strongly suggest that the blockade of PDK1/PLCγ1 interaction by 2-*O*-Bn-InsP_5_ is sufficient to inhibit cell invasion (Fig. 7). However, our results do not rule out the possibility that 2-*O*-Bn-InsP_5_ might act via other PDK1 targets beside PLCγ1. Therefore, it remains to be determined whether 2-*O*-Bn-InsP_5_ could affect these or other biological functions also by targeting additional effectors in addition to PDK1- or PLCγ1-dependent effectors. Altogether our data show for the first time that the PDK1/PLCγ1 interaction is pharmacologically targetable and that its inhibition may have anti-metastatic effects *in vivo.* Therefore, 2-*O*-Bn-InsP_5_ could potentially be a promising anti-cancer lead compound to design novel anti-metastatic drugs. Allosteric compounds have emerged as an attractive new class of small molecule inhibitors in cancer. Due to their specific mode of interaction that does not involve competition with ATP at the active site, they have been shown to be highly selective. We were among the first to suggest that activation of proteins involved in cell growth and tumourigenesis could be inhibited by interfering with their translocation to the plasma membrane^24^. In particular we showed that cytoplasmatic inositol phosphates could compete with PtdIns(3,4,5)*P*_*3*_ for the binding to AKT PH domain preventing its translocation to the plasma membrane and activation^24^ thus representing an important alternative to the use of inhibitors directly targeting the catalytic domain^24^. Recent work has reinforced the idea that small molecule inhibitors can act by interfering with the localization of proteins with key roles in cancer progression^25^,^26^. For instance, although the cancer-associated protein KRAS had long been considered undruggable, a novel strategy was recently developed based on the indirect inhibition of its membrane localization^26^,^27^. In this respect results from our current work provide further support to the conclusion that inhibition of protein membrane translocation can represent a useful alternative strategy to block protein activation and ultimately processes associated with tumorigenesis. By binding to PDK1 PH domain, the allosteric inhibitor 2-*O*-Bn-InsP_5_ inhibits PDK1 and PLCγ1 membrane localisation and the PDK1/PLCγ1 complex formation required for full PLCγ1 activation. Thus, the allosteric PDK1 inhibitor 2-*O*-Bn-InsP_5_ targets PDK1 activity with a different mechanism of action than currently available ATP competitive inhibitors like GSK2334470. Because 2-*O*-Bn-InsP_5_ interferes with membrane localisation of PDK1 and PLCγ1 (Fig. 3) it could be speculated that 2-*O*-Bn-InsP_5_ could selectively inhibit activation of PDK1 substrates by interfering with PDK1 (and its substrates) localisation to the plasma membrane. Since PDK1 has been shown to be a master regulator at the hub of many downstream signalling pathways, the effect of an allosteric inhibitor that would interfere with specific downstream substrates activation by modulating PDK1 spatial localization would be of great value allowing for the selective inhibition of some of the PDK1 signalling over others. Taken together, our findings show that 2-*O*-Bn-InsP_5_ could lead to the development of distinct more selective (hence less toxic) therapeutic strategies by targeting specifically the PDK1/PLCγ1 complex formation at the plasma membrane and its downstream pathways that may lead to the development of novel drugs capable of selectively inhibiting the invasive potential of cancer cells.

## Materials and Methods

### Reagents and antibodies

Chemicals were purchased from Sigma-Aldrich, UK. Human PDGF was purchased from R&D Systems. Recombinant EGF was purchased from Peprotech, UK. Antibodies were purchased as follows: pAKT (Thr308), pAKT (Ser473), pPLCγ1 (Y783), total AKT and PDK1, pERK1/2 T202/Y204 and total ERK1/2 from Cell Signaling Technology, GAPDH from Abcam, PLCγ1 from Santa Cruz Biotechnology. Glass-bottomed 35-mm dishes were obtained from MatTek Corporation. InsP_5_ and 2-*O*-Bn-InsP_5_ were synthesised as previously reported^21^,^28^. Each compound was purified to homogeneity by ion-exchange chromatography on Q-Sepharose Fast Flow resin and used as the triethylammonium salt, which was fully characterized by ^31^P and ^1^H spectroscopy and accurately quantified by total phosphate assay.

### Cell Culture and Transfections

MDA-MB-231, MDA-MB-435, 4T1 and NIH 3T3 cells lines were obtained from the American Type Culture Collection (Manassas, VA, USA). TSA were provided by Dr. P. Lollini, Istituto di Cancerologia, University of Bologna, Italy, and A375M were provided by Dr Daniele Bergamaschi, QMUL, UK. MDA-MB-231 were cultured in DMEM (Life Technologies, UK); MDA-MB-435, 4T1, TSA and A375M were cultured in RPMI 1640 (Life Technologies or PAA, UK) supplemented with 10% (v/v) Foetal Bovine Serum (FBS), Sodium Pyruvate 1% (v/v), 1X L-glutamine/penicillin/streptomycin and 0.1% gentamycin (v/v). NIH 3T3 cells were maintained in DMEM containing 10% donor calf serum. NIH 3T3 cells were transfected with 1 μg of DNA encoding for eGFP-Akt, eGFP-Akt-mRFP or eGFP-PDK1^29^ using Lipofectamine LTX with PLUS reagent (Life Technologies) in OptiMEM containing GlutaMAX (Life Technologies), as recommended by the manufacturer. MDA-MB-231 cells were co-transfected with pOZ-PDK1 and PRK5-PLCγ1 using Lipofectamine (Life Technologies) according to the manufacturer’s instructions. siRNA transfection was performed by using Hiperfect (Qiagen, UK) for A375M as previously described^17^. SMARTpool siRNA targeting PLCγ1 were purchased from Dharmacon, USA whilst the non-targeting siRNA (*Silencer*^®^ Negative Control #1 siRNA) was purchased from Applied Biosystems.

### Cell migration and Matrigel invasion assay

MDA-MB-231, MDA-MB-435, TSA, 4T1 and A375M cell lines were serum starved overnight before the experiment. Cell migration and invasion assays were performed as described^17^,^30^. Briefly, cells were left untreated or pre-treated with 50 μM of 2-*O*-Bn-InsP_5_ for 30 minutes before being detached, counted and plated on inserts. For migration assays, a suspension of 10,000 cells in 150 μl was homogenously added in the upper chamber and the lower chamber was filled with RPMI or DMEM containing 0.5% BSA. Cells were allowed to migrate for 4 hours at 37 °C, 5% CO_2_ in the absence or presence of the inhibitor. For invasion assay, Matrigel pre-coated inserts (8.0 μm pores, 10 mm diameter, BD Bioscience, UK) were used. Inserts were re-hydrated for 1 hour in DMEM or RPMI 1640 according to the cell type before the experiment. A suspension of 10,000 cells (60,000 for MDA-MB-435) in 500 μl was homogenously added in the upper chamber and the lower chamber was filled with RPMI or DMEM containing 10% FBS. Cells were allowed to invade for 36 hours at 37 °C, 5% CO_2_ in the absence or presence of the inhibitor. Cells that did not migrate or invade were removed using a cotton bud whereas cells that had migrated or invaded were fixed with paraformaldehyde and stained with 0.1% crystal violet solution for 10 minutes. A Leica phase-light microscope using a 10X magnitude objective was used for manual counting. A minimum of five fields was counted per insert. Each experiment was performed in duplicate and the average of cells/fields was calculated.

### Intracellular Calcium Measurement

MDA-MB-231 and MDA-MB-435 were seeded on bottom-glassed chambers (Labtek, UK) and serum starved overnight. Cells were then incubated with HBSS (Life Technologies) containing 0.5% BSA, 2 mM CaCl_2_, 4 μM Fluo-4-AM (Life Technologies) for 45 minutes at 37 °C, washed twice in HBSS 0.5% BSA 2mM CaCl_2_ and left in the same solution for 30 minutes for de-esterification of the Fluo-4-AM dye. Where indicated 50 μM InsP_5_ or 50 μM 2-*O*-Bn-InsP_5_ were added during the de-esterification step for 30 minutes. Fluorescence was measured using the LSM 510 inverted confocal microscope equipped with a chamber for live imaging at 37 °C supplied with 5% CO_2_ using a 20X objective. After recording basal fluorescence, cells were then stimulated with EGF (20 ng/ml) and fluorescence was measured for 6 minutes before further stimulation with 1 mM ATP (Sigma, UK). Thirty cells were selected and variation of fluorescence intensity was analysed for each sample.

### Lysis conditions and Western blotting analysis

After stimulation or treatment, NIH 3T3 were lysed for 5 min on ice in Lysis buffer [20 mM Tris-HCl (pH 7.4), 150 mM NaCl, 100 mM NaF, 10 mM Na_4_P_2_O_7_, and 10 mM EDTA supplemented with one complete protease inhibitor cocktail tablet (Roche) and 1% Triton X-100]. To terminate the reaction, SDS Sample buffer [125 mM Tris- HCl (pH 6.8), 6% SDS, 20% glycerol, and 0.02% bromophenol blue supplemented with 10% β-mercaptoethanol] was added and the samples were boiled for 5 min. The proteins were separated on a NuPAGE 4 to 12% Bis-Tris Gel (Life Technologies) and transferred to a polyvinylidene difluoride (PVDF) membrane (Immobilon FL, Millipore). For protein detection with the Odyssey Infrared Reader (LI-COR), membranes were incubated in blocking buffer (LI-COR) for 1 h, and incubated for 24 h at 4 °C with antibodies against pAKT (Thr308) together with total AKT, or pAKT (Ser473) together with total AKT (each antibody diluted in blocking buffer at 1:1000). The infrared dye-conjugated secondary antibodies IRDye 800CW and 680LT (Rockland) were used at a 1:5000 dilution in blocking buffer for 1 h.

MDA-MB-231 were lysed in lysis buffer containing 50 mM Tris-HCl (pH 7.4), 5 mM EDTA, 0.1% NP40, 250 mM NaCl, and proteases inhibitors cocktail 2 (Sigma-Aldrich, UK). Lysates were then centrifuged at 13,000 rpm for 10 min at 4 °C. Proteins were separated by SDS/PAGE and transferred to nitrocellulose. Membranes were incubated with a solution of 5% milk (w/v) and then incubated overnight with the diluted primary antibodies [pPLCγ1(Y783) or GAPDH]. The appropriate peroxidase-conjugated secondary antibodies (Sigma-Aldrich, UK) were used and proteins were detected by enhanced chemi-luminescence reaction (GE Healthcare, UK).

### Phosphokinase antibody array

Following overnight starvation, MDA-MB-231 cells were left untreated or pre-treated with 50 μM 2-*O*-Bn-InsP_5_ and then stimulated with 50 ng/ml EGF for 10 min in the presence or absence of the inhibitor. Cells were lysed as described above and phosphokinase antibody array was performed according to the manufacturer’s instructions (R&D Systems).

### Co-immunoprecipitation

Four sets of three 4-cm Petri dishes of MDA-MB231 (two sets treated for one hour with 50 μM 2-*O*-Bn-InsP_5_ and two sets treated with vehicle only) were stimulated with 50 ng/ml EGF for the indicated time point, then lysed in lysis buffer (50 mM Tris pH 8.0, 50 mM KCl, 1% NP-40) containing protease and phosphatases inhibitors (Sigma-Aldrich, UK). 3 μg of anti-PLCγ1 antibody (Santa Cruz Biotechnology, USA) or control mouse IgG were immobilised onto 30 μl of magnetic protein G Dynabeads (Life Technologies) using the cross-linker Bis(sulfosuccinimidyl)suberate (BS3). Briefly, Dynabeads were incubated for 1 hour at RT with anti-PLCγ1 antibody or control mouse IgG and then incubated for 30 minutes with BS3 conjugation buffer (5 mM BS3, 20 mM Sodium Phosphate, 0.15 M NaCl pH. 8.0). The crosslinking reaction was quenched by incubating the Dynabeads with BS3 quenching buffer (1 M Tris HCl pH 7.5). Dynabeads were then resuspended in lysis buffer and used for immunoprecipitation. Lysates were cleared by centrifugation at 10,000 *g* for 3 minutes at +4 °C. 2.5 mg of protein lysates were mixed with 30 μl of Dynabeads previously cross-linked to anti-PLCγ1 antibody (Santa Cruz Biotechnology, USA) or control mouse IgG, and incubated overnight at + 4 °C. Beads were collected with a Dynabead magnet, washed three times with lysis buffer on a rotating wheel at 4 °C for 5 min, and resuspended in 50 μl Laemmli sample buffer for SDS-PAGE and immunoblotting.

### Confocal Microscopy Analysis

MDA-MB-231 cells were co-transfected with PRK5-PLCγ1 and pOZ-PDK1. Twentyfour hours after transfection cells were serum deprived overnight. The following day, cells were left untreated or treated with 50 μM 2-*O*-Bn-InsP_5_ for 30 minutes, stimulated with serum free DMEM containing EGF (50 ng/ml) in the presence or absence of 50 μM 2-*O*-Bn-InsP_5_ for the indicated times and then fixed in paraformaldehyde 4% (v/v) for 30 minutes at room temperature (RT). Cells were permeabilised with PBS containing 0.25% Triton-X-100 for 2.5 minutes at RT. Unspecific staining was prevented by blocking the coverslips with a solution of PBS/0.1% BSA for 30 minutes at RT. Coverslips were then incubated overnight with primary antibodies (anti-mouse-PLCγ1 and anti-rabbit-PDK1 diluted 1:50 in PBS/0.1% BSA). Coverslips were then washed 3X with PBS/0.1% BSA, incubated with secondary antibodies anti-mouse-Alexa488 (Life Technologies) and anti-rabbit Alexa555 (Life Technologies) and analysed using a Carl Zeiss LSM 510 Meta confocal microscope using a Zeiss plan apochromat 63× 1.4 NA(oil) lens.

### Translocation analysis

MDA-MB-231 co-transfected with PRK5-PLCγ1 and pOZ-PDK1 were treated as for confocal microscopy analysis and coverslips were analysed using a Leica Epi-fluorescence microscope. MDA-MB-231 untreated or treated with 2-*O*-Bn-InsP_5_ (50 μM) were stimulated with EGF (50 ng/ml) in the absence or presence of 2-*O*-Bn-InsP_5_ (50 μM). The number of cells displaying translocation of exogenous PLCγ1 and PDK1 to the membrane was manually counted and expressed as percentage. A minimum of 100 cells were counted for each condition per experiment in three independent experiments.

### Time resolved FRET by multiple frequency domain FLIM

Cells were seeded at 150,000 per 35-mm glass-bottomed tissue culture dish (MatTek) and transfected as described earlier. After stimulation with PDGF (30 ng/ml) for 5 min, the cells were washed with PBS and then fixed in PBS containing 4% paraformaldehyde for 10 min. The dishes were washed with PBS and then mounted with Mowiol supplemented with 2.5% (w/v) 1,4-diazabicyclo[2.2.2]octane (DABCO) at 4 °C. Time-resolved FRET was used to determine the variations in AKT conformation. The lifetime of the eGFP donor chromophore expressed as a fusion protein in the cells was acquired using the Lambert Instruments multiple frequency domain Fluorescence Lifetime Imaging Microscope (FLIM). The FRET efficiency (E_f_) for all the pixels was calculated for each cell as: E_f_ = 1−(τ_D+A_/τ_D_), τ_D_ being the phase lifetime of the donor (eGFP-AKT) and τ_D+A_ the phase lifetime of the donor in presence of an acceptor (eGFP-AKT-mRFP). Details of the frequency domain FLIM, operation, acquisition parameters and data analysis have been detailed previously^31^.

### Steady state FRET by Acceptor Photo-bleaching

Cells were treated as for confocal microscopy analysis. Cells were imaged with excitation *λ* = 488 nm and *λ* = 543 laser line and emission spectra were collected respectively in two different channels of the PMT detector. Briefly, cells overexpressing PLCγ1 or PDK1 or co-expressing PLCγ1 and PDK1 were co-stained with Alexa-488 and Alexa-555 conjugated antibodies. The FRET efficiency was measured by acceptor photo-bleaching, as previously described^17^,^32^. A selected area of the cell was repeatedly photobleached with *λ* = 543 nm laser line at full power for 1 minute and FRET efficiency was measured as the increase (or dequenching) of donor fluorescence after photo-bleaching in the selected area. All fluorescence measurements were performed in MetaMorph software (Molecular Devices Inc., USA). Reference spectra were generated from coverslips incubated separately with the two Alexa-conjugated secondary antibodies alone in order to generate single positive sample and all data were corrected for cross-talk and background fluorescence. Percentage of FRET was calculated measuring donor fluorescence of 10 cells before and after acceptor bleaching.

### Preparation of DNA constructs

The PH domains of human AKT2 [amino-acids 1-116] and human PDK1 [amino-acids 411-552] were PCR amplified using the oligos AKT2 PH-s: 5-g gat cct atg aat gag gtg tct gtc atc aaa gaa ggc-3 and AKT2 PH-a: 5-gaattc tca gcc tgg ggc ccg ctg ctt gag gc-3 and PDK1 PH-s: 5-g gat cct atg aac ata gag cag tac att cac gat-3 and PDK1 PH-a: 5-gaattc tca gtc cgg gtg gct ctg gta tcg ctg-3 respectively. The PCR products were subcloned BamHI-EcoRI into the vector pTriEX6-HIS-GST-3C, a modified version of the Novagen vector pTriEX-6 to create pTriEX6-GST-PH AKT2 and pTriEX6-GST-PH PDK1.

### Preparation of the recombinant PH domains

BL21 (DE3) (100 μl) were electroporated with 500 ng of either pTriEX6-GST-PH AKT2 or pTriEX6-GST-PH PDK1 plasmid DNA, recovered in 200 μl of SOC medium and plated on ampicillin plates. A colony was picked and grown overnight in 5 ml L-Broth supplemented with 100 μg/ml of ampicillin at 37 °C in a shaking incubator. This starter culture was used to inoculate 2 × 200 ml L-Broth/ampicillin and allowed to grow at 37 °C with shaking. When the OD_600nm_ reached approximately 0.5 the expression of the proteins was induced by addition of 1 mM IPTG. Cultures were allowed to grow overnight at 20 °C in a shaking incubator.

The bacteria from each culture were pelleted by centrifugation and then lysed at 4 °C in 2 × 10 ml of lysis buffer comprised of Buffer A (50 mM Tris-HCl pH7.5; 150 mM NaCl; 1 mM EDTA; 5% glycerol) supplemented with 1% Triton X-100, 1 mM DTT, 10 mM β−Glycerophosphate, 1 mM NaF, 10 mM Benzamidine and complete protease inhibitor cocktail (Roche). The re-suspended bacteria were sonicated (3 × 10 s at an amplitude of 10 microns at 4 °C) and centrifuged at 20,000 g for 10 min at 4 °C. The supernatant was subsequently added to glutathione-sepharose beads (1 ml bed volume) and incubated for 2 h. After extensive washing with Buffer A + 1 mM DTT the beads were re-suspended in 500 μl of the same buffer and the PH domains cleaved from the beads by addition of 3C protease. Cleavage was allowed to occur overnight at 4 °C. The 1 ml total volume of cleaved PH domains were concentrated to 500 μl using a Vivaspin 5,000 Da cut off concentrator and applied to a S75 size exclusion column equilibrated in the ITC Buffer comprising of Buffer A + 0.5 mM TCEP.

### Isothermal Titration Calorimetry Experiments

The purified PH domains of PDK1 and AKT2 were adjusted to 45 μM and 48 μM respectively for the ITC experiments. The inhibitor 2-*O*-Bn-InsP_5_ was diluted with ITC buffer (see above) to a 10 fold higher concentration than the individual PH domains. ITC titrations were performed on a VP-ITC200 MicroCalorimeter (MicroCal) at 25 °C (unless otherwise stated) with 15 × 3 μL injections of 2-*O*-Bn-IP5 into PH domain located in the cell. Subtraction of the heats of dilution, integration of the raw data and subsequent fitting to a one site binding model was carried out using the Origin (version 7) software supplied with the instrument.

### Zebrafish xenotransplant

Zebrafish (*Danio rerio*) were handled in compliance with local animal care regulations and standard protocols. All experiments were performed in accordance with relevant guidelines and regulations and were subject to local ethics approval and done in accordance with the Animals (Experimental Procedures) Act, 1986, under license from the United Kingdom Home Office. The QMUL Ethics Committee approved our experiments and all procedures are done in accordance with the UK Home Office Licensing (PPL 70/7452). Fish were kept at 28 °C in aquaria with day/night light cycles (10-hour dark/14-hour light periods). The developing embryos were kept in an incubator at constant temperature. At 48 h post-fertilization, zebrafish embryos were dechorionated with sharp forceps (Dumont Tweezers #5, World Precision Instruments Ltd, UK) and anesthetized with 0.04  mg/ml of tricaine (MS-222, Sigma). Anesthetized embryos were then transferred into a 1% agarose gel for microinjection.

For injection into the heart^22^, 50–100 cells, manually counted, were injected above the ventral end of the duct of Cuvier where it opens into the heart using a manual injector (Picospritzer III, Parker Hannifin Instruments). After injection, the zebrafish embryos were briefly imaged using a Leica SPE confocal microscope. Embryos displaying a similar number of injected cells were selected and divided into a control (untreated) group and treated group (incubated in marine salt water supplemented with 100 μM 2-*O*-Bn-InsP_5_). Embryos were then immediately transferred into an incubator set at 35 °C to compromise between the optimal temperature requirements for fish and mammalian cells. Three days after injection zebrafish embryos were immunostained with rabbit anti-GFP (MBL) and chicken anti-RFP (MBL) followed by staining with Alexa-conjugated goat anti rabbit and goat anti chicken antibodies (Life Technologies) and imaged with a Zeiss LSM 710 Scanning Confocal Microscope using a 20x objective. 3D-rendered models of zebrafish embryos were generated with Imaris (Bitplan AG) using tiled z-stacks of 15–20 confocal slices. Dimensional analysis of metastases was carried out using the surface rendering function of Imaris based on the GFP signal throughout the tiled z-scan series. GFP signal detected within the vessel was excluded by generating a mask based on the mCherry signal within the blood vessels. Autofluorescence from the yolk sac and signal from the intestine was excluded. The number and the volume of metastasis were measured in n = 6 control and n = 10 treated embryos from 2 independent experiments. Experiments were discarded when the survival rate of the control group was <90%. Alternatively ~300 cells were resuspended in PBS and 1–5 nL of tumour cells solution were injected into the perivitelline cavity of each embryo^33^ using a manual injector (Picospritzer III, Parker Hannifin Instruments). After injection, the fish embryos were immediately transferred into 35 °C incubator and examined every day for monitoring tumor dissemination using a fluorescent microscope. Three separate experiments were performed. Counting of disseminated cells was done using a Zeiss Axioplan epifluorescence microscope and disseminated cells counted under high magnification.

### Zebrafish locomotor activity assay

We assayed 6dpf untreated embryos (n = 15), or treated with 2-*O*-Bn-InsP_5_ (n = 16) for their locomotor activity. Larvae were raised at 28 °C on a 14/10 h light/dark cycle. Embryos were fed on day 5, and morning of day 6. Two hours before the behavioural procedure, embryos were transferred to a clean plate with no food, and were then moved to the behavioural room. For each trial, 4 embryos were transferred to a tank (11.2 ×7.5 × 2.0 cm) containing 4 insets (6.8 × 2.2 × 2.0 cm) with opaque walls, each with 15 mL of water. The tank was placed inside a Zebrabox (ViewPoint Life Sciences) that was continuously illuminated with infrared and white lights. The swimming behaviour of each fish was monitored for 10 minutes using an automated video-tracking system (Zebralab, ViewPoint Life Sciences), and the movement of each larva was recorded using the Zebralab tracking quantization mode. The Zebralab tracking quantification thresholds were set in the following way: detection threshold, 120 (color transparent); movement threshold: high-speed, 6.0 mm/sec and inactivity, 2.0 mm/sec; bin size, 60 s. Red and green tracking lines in Fig. 6G correspond to high-speed and slow-speed trajectories, respectively. Locomotor activity was quantified based on the total distance covered by each fish.

### Statistical analysis

A nonparametric Mann-Whitney test was used to compare the medians of the two data sets for the FLIM data with GraphPad Prism software. To interpret the distribution of data, we used box and whiskers plots. The box and whiskers plot is a histogram-like method for displaying upper and lower quartiles and maximum and minimum values in addition to the median. Each red symbol represents the FRET efficiency of one cell. For the Zebrafish tumor experiments and the locomotor activity assay a two-tailed, unequal variance Student’s *t*-test was used.

In all the other experiments statistical analyses was performed using the one-tailed, paired Student’s *t*-test.

## Additional Information

**How to cite this article**: Raimondi, C. *et al*. A Small Molecule Inhibitor of PDK1/PLCγ1 Interaction Blocks Breast and Melanoma Cancer Cell Invasion. *Sci. Rep.* **6**, 26142; doi: 10.1038/srep26142 (2016).

## Supplementary Material

Supplementary Information

## Figures and Tables

**Table 1:** Summary of the thermodynamic parameters for the binding of 2-*O*-Bn-InsP_5_ to the PH domain of PDK1 at 25 °C.

**n (sites)**	**H**_**obs**_ **(kcal/mol)**	**TΔS (kcal/mol)**	**G (kcal/mol)**	**D (nM)**
0.91	−4.897	−0.289	−5.186	92
0.94	−3.769	−0.293	−4.062	78
0.89	−3.816	−0.291	−4.107	174
0.84	−4.005	−0.291	−4.296	140
0.90 ± 0.04	−4.122 ± 0.527	−0.291 ± 0.002	−4.413 ± 0.525	109 ± 44
