# Supplementary material for: A Small Molecule Inhibitor of PDK1/PLCγ1 Interaction Blocks Breast and Melanoma Cancer Cell Invasion
Source: Sci Rep. 2016 May 20;6:26142. doi: 10.1038/srep26142 (PMC4873738; doi:10.1038/srep26142)
Supplement: Supplementary Information [file srep26142-s1.doc]

**A Small Molecule Inhibitor of PDK1/PLCγ1 Interaction Blocks Breast and Melanoma Cancer Cell Invasion**

Claudio Raimondi^1,†^ , Veronique Calleja^2,#^, Riccardo Ferro^1^, Alessandro Fantin^3^, Andrew M. Riley^4^, Barry V. L. Potter^4,5^, Caroline H. Brennan^6^,

Tania Maffucci^1^, Banafshé Larijani^2,&^ and Marco Falasca^1,7,*^

**Supplementary Information**

**Supplementary Figure 1**

**
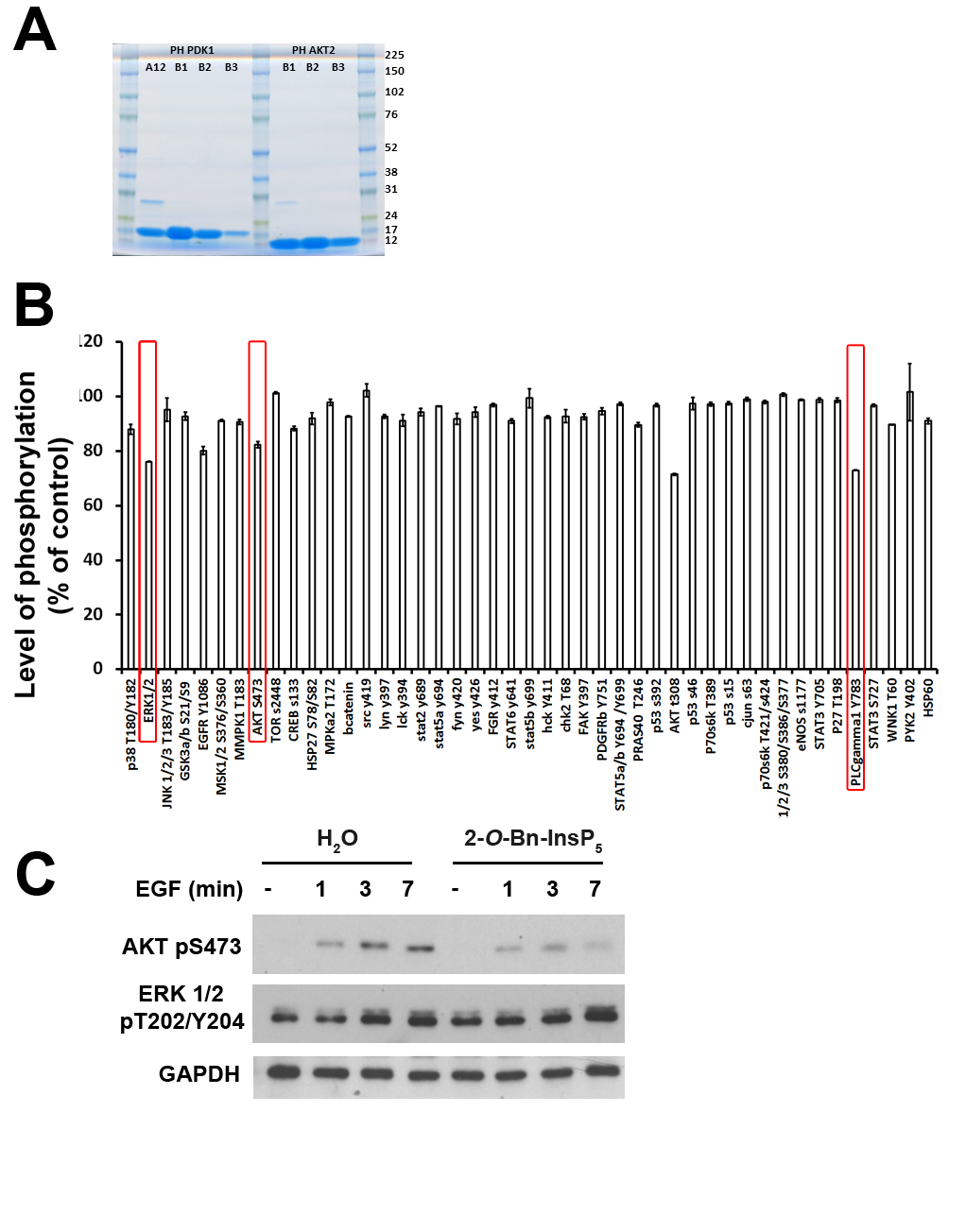
**

**Supplementary Figure 1.** (A) Assessing the purity of the PDK1 and AKT2 PH domains. The purity of the PH domains after S75 size exclusion chromatography was assessed by SDS-PAGE using a 4-12% polyacrylamide gel and visualised with the Gelcode protein staining solution. The fractions analysed correspond to peaks on the resulting chromatogram (A12-B3 and B1-B3 indicate fraction number for the PH domain of PDK1 and AKT2 respectively). (B) Results from phosphokinase antibody array performed on lysates form MDA-MB-231 treated with 50 μM 2-*O*-Bn-InsP_5_ or vehicle alone and stimulated with EGF 50ng/ml for 3 minutes. (C) Representative Western blot of EGF-induced AKT serine 473 and ERK1/2 T202/Y204 phosphorylation in MDA-MB-231 untreated or treated with 50 μM 2-*O*-Bn-InsP_5_.

**Supplementary Figure 2**

**
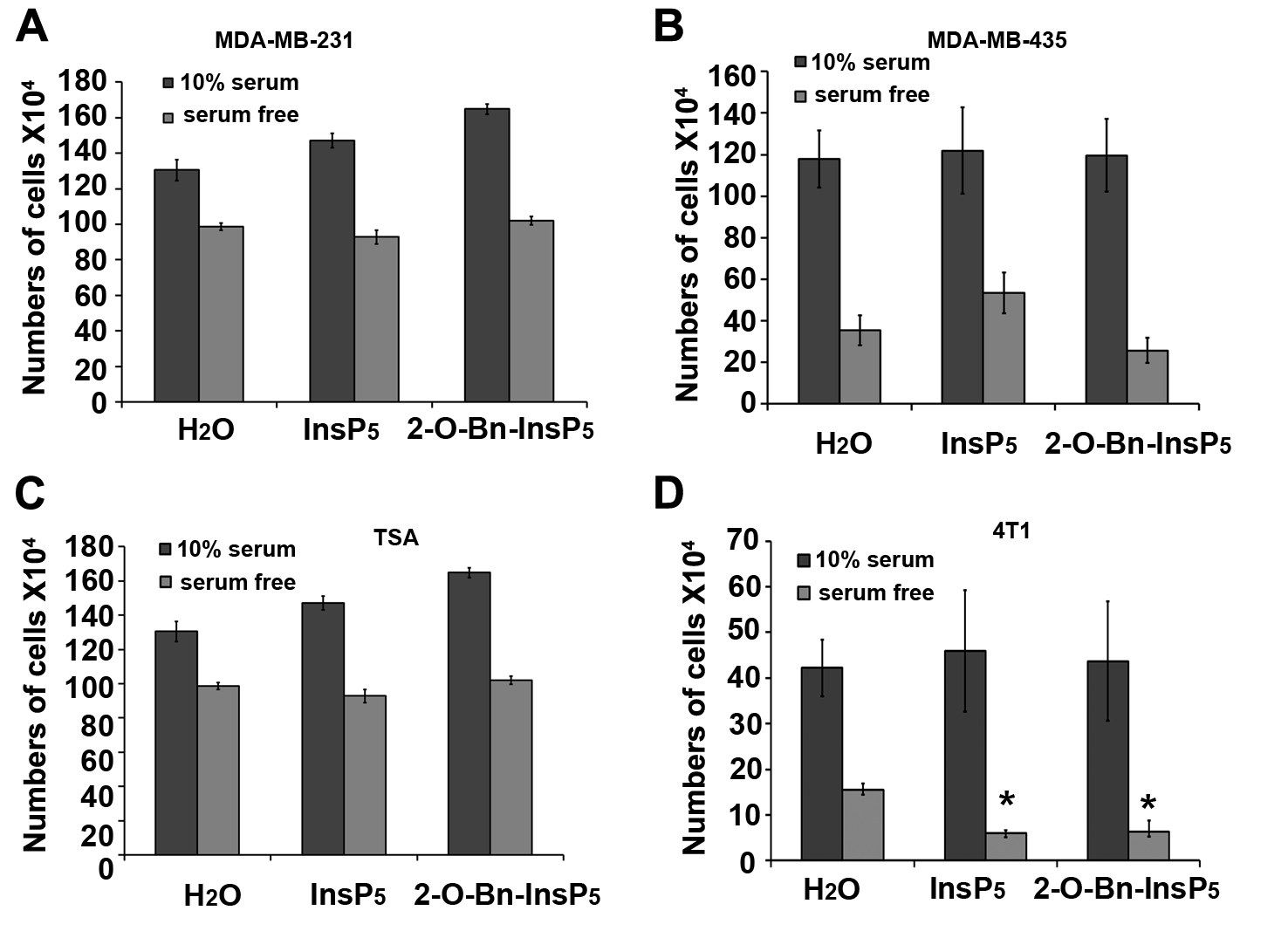
**

**Supplementary Figure 2.** Cell counting experiment assessing cell proliferation (10% serum) and survival (serum free condition) in MDA-MB-231 (A), MDA-MB-435 (B), TSA (C) and 4T1 cell lines (D) after 72 hours of treatment with 2-*O*-Bn-InsP_5_ and InsP_5_ (50 μM). Data are means ±SEM of values obtained from 3 independent experiments in duplicate. *p<0.05.
